# Supplementary material for: The Different Effects of Substrates and Nucleotides on the Complex Formation of ABC Transporters
Source: Structure. 2019 Apr 2;27(4):651–659.e3. doi: 10.1016/j.str.2019.01.010 (PMC6453779; doi:10.1016/j.str.2019.01.010)
Supplement: Document S1. Figures S1–S5 [file mmc1.pdf]

Structure, Volume *27*

Supplemental Information

The Different Effects of Substrates and Nucleotides on the Complex Formation of ABC Transporters

Francesco Fiorentino, Jani Reddy Bolla, Shahid Mehmood, and Carol V. Robinson

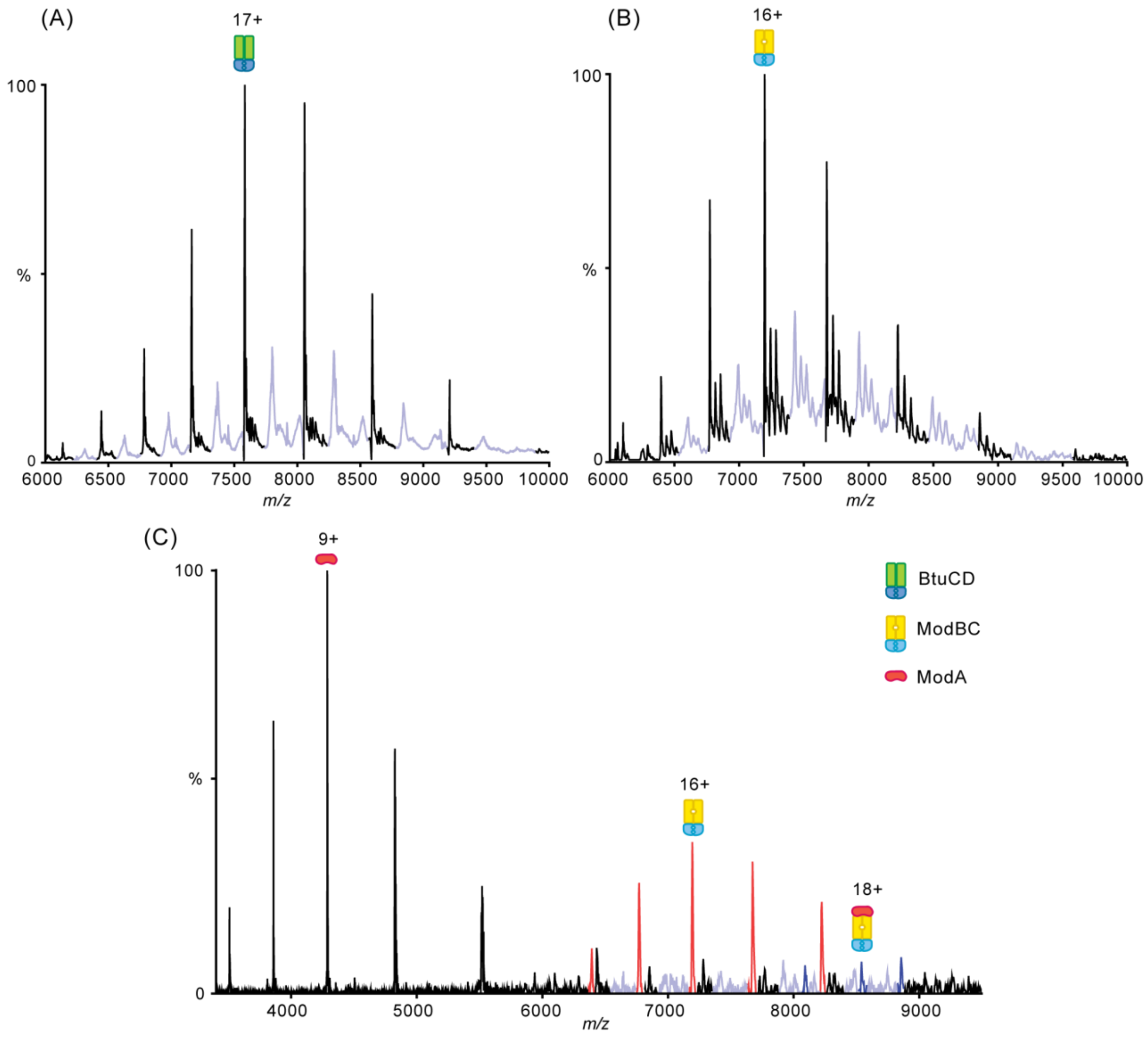

Figure S1. Mass spectra of uncomplexed BtuCD and ModBC, Related to Figure 1. (A) Mass spectrum of BtuCD in 0.50% (w/v) C_8E_4 reveals a charge state series consistent with the tetrameric transporter with different lipid-bound species, mostly LPS (light blue peaks). (B) Mass spectrum of ModBC in 0.50% (w/v) C_8E_4 reveals a charge state series consistent with the tetrameric transporter bound to different lipid species, mainly LPS (light blue peaks). (C) Mass spectrum of ModBC and ModA acquired in the absence of molybdate shows only a low population of the full ModBC-A complex (blue peaks).

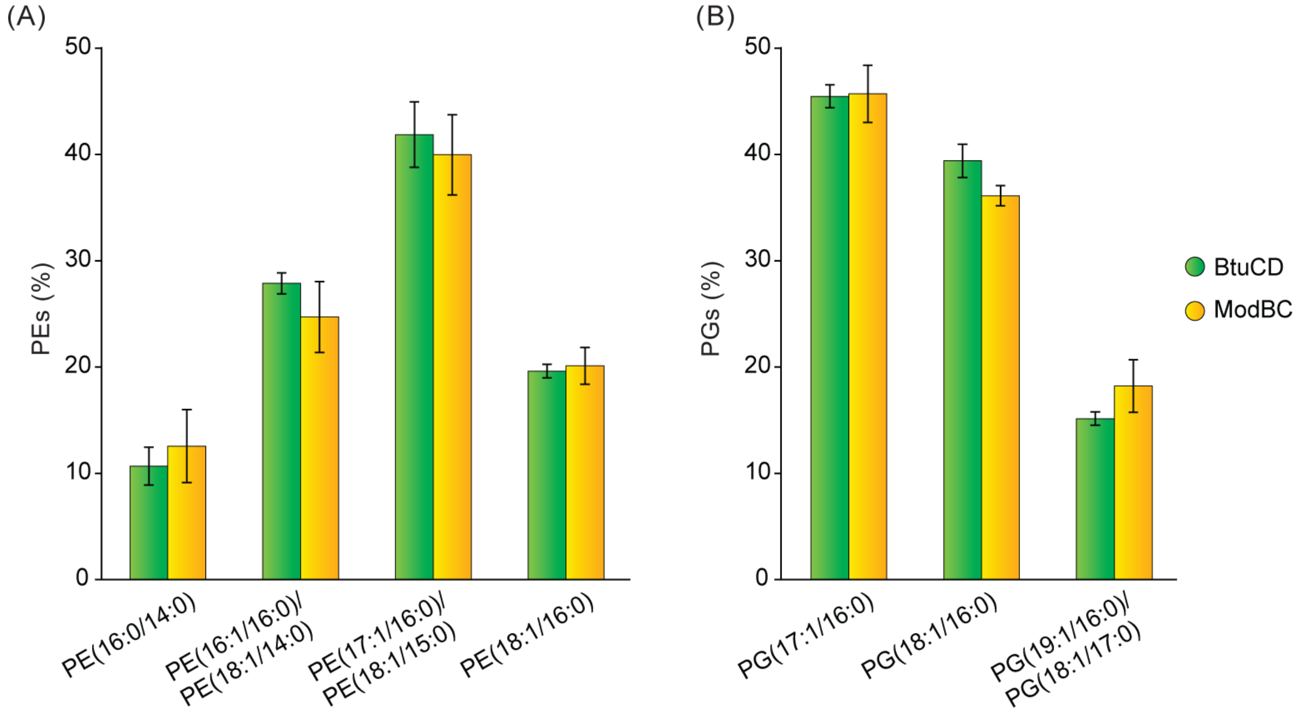

Figure S2. Lipidomics analysis for BtuCD (green) and ModBC (yellow), Related to Figure 1. (A) Relative abundance of phosphatidylethanolamines (PEs). (B) Relative abundance of phosphatidylglycerols (PGs). Error bars represent standard deviations (*n*=3).

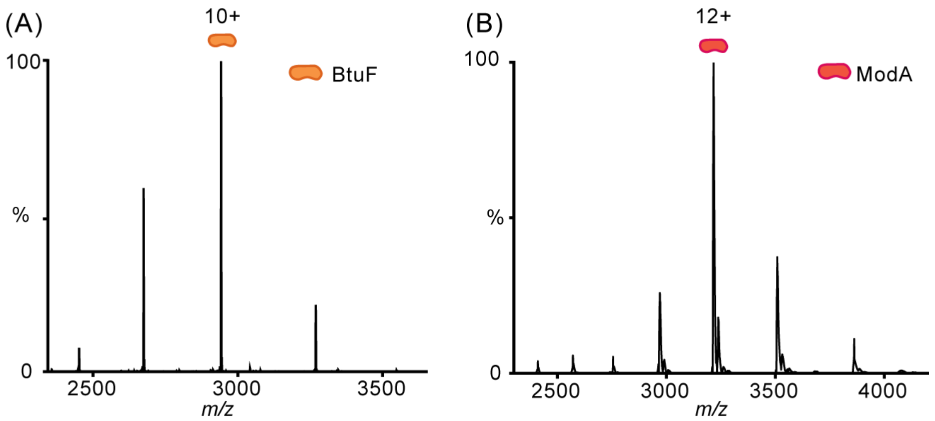

Figure S3. Mass spectra of BtuF (A) and ModA (B), Related to Figure 1. Both proteins were separately purified and analysed in the absence of any detergent. Theoretical and observed masses and recorded in Table 1.

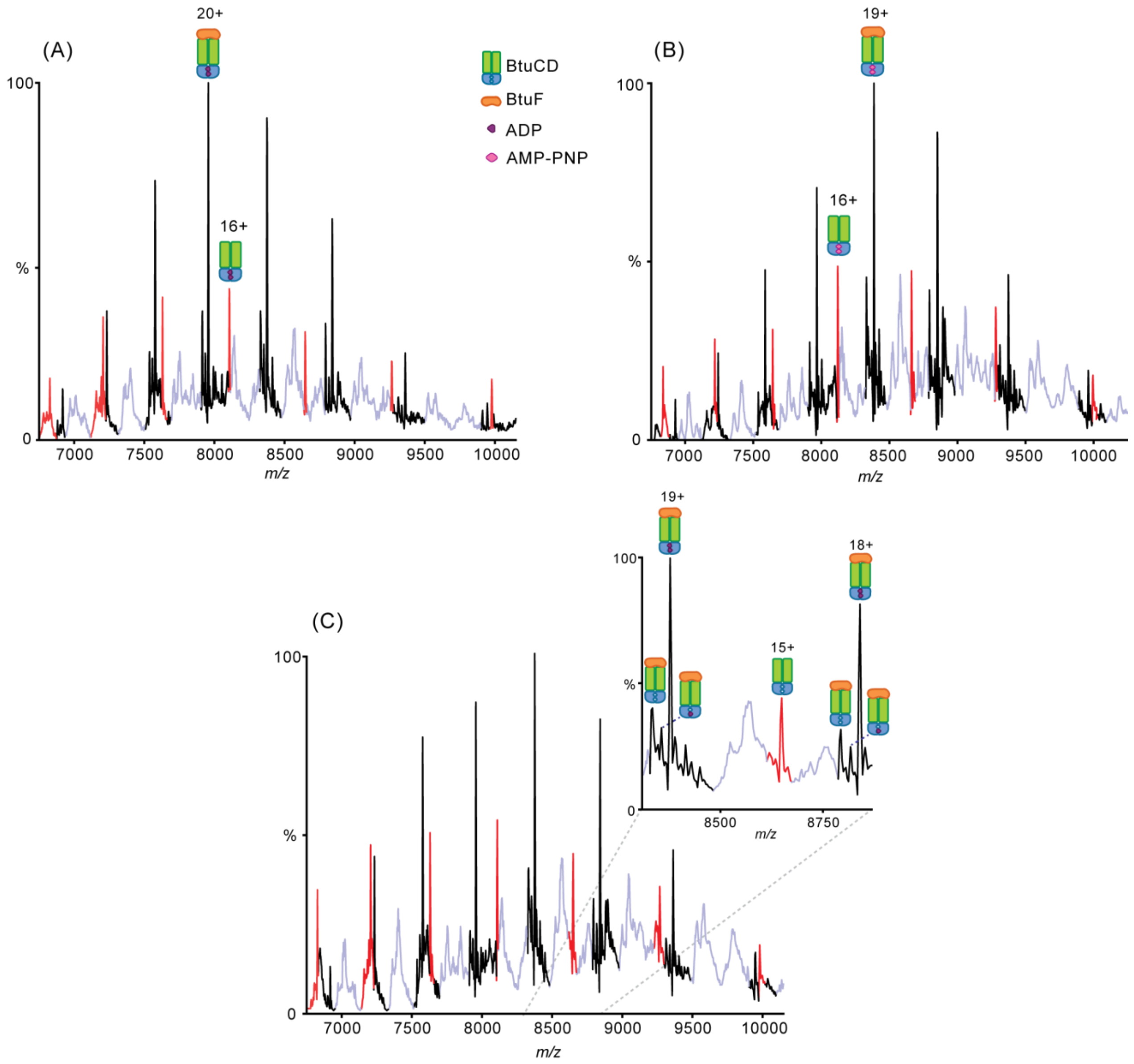

Figure S4. Nucleotides destabilise BtuCD-BtuF interaction, Related to Figure 3. (A) Mass spectrum of BtuCD-F recorded after addition of 5 mM ATP to BtuCD before buffer exchange. ADP-bound species are detected with binding indicative of cooperativity along with an increase in the intensity of the BtuCD charge states (red peaks). (B) Mass spectrum of BtuCD-F recorded after addition of 5 mM AMP-PNP to BtuCD before buffer exchange. Also in this case, AMP-PNP-bound species are detected with binding indicative of cooperativity along with an increase in the intensity of the BtuCD charge states (red peaks). (C) Mass spectrum of BtuCD-F recorded after addition of 5 mM ATP to BtuCD and 2mM vitamin B_{12} to BtuF before buffer exchange. The spectrum shows a further increase in the BtuCD charge states evident of a synergistic effect upon the transport of B_{12} and the binding of nucleotides (red peaks).

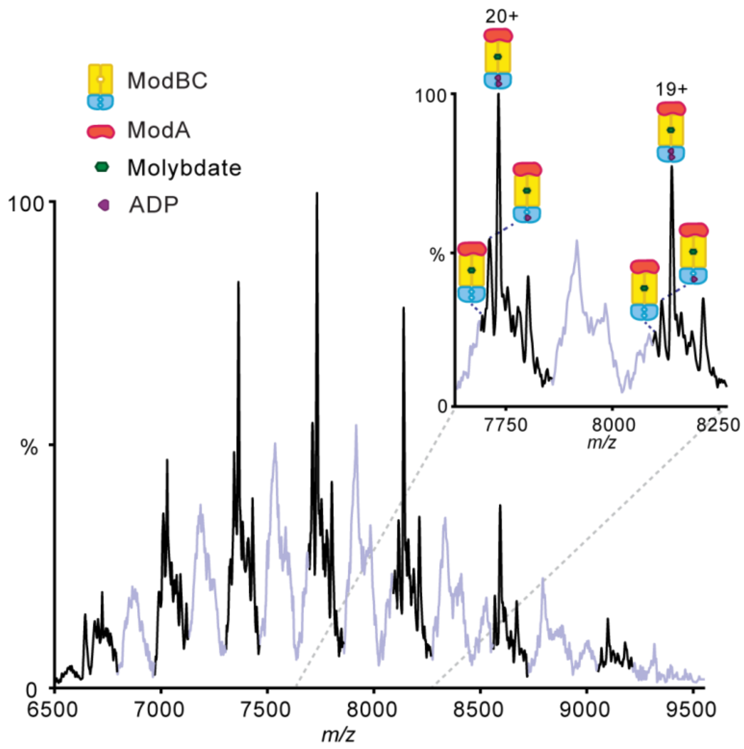

Figure S5. Mass spectrum of ADP-bound form of ModBC-A, Related to Figure 3. Mass spectrum of ModBC-A following the addition of 10 µM ATP to ModBC after buffer exchange.
