# Supplementary material for: The Different Effects of Substrates and Nucleotides on the Complex Formation of ABC Transporters
Source: Structure. 2019 Apr 2;27(4):651–659.e3. doi: 10.1016/j.str.2019.01.010 (PMC6453779; doi:10.1016/j.str.2019.01.010)
Supplement: Document S2. Article plus Supplemental Information [file mmc2.pdf]

Article

Structure

The Different Effects of Substrates and Nucleotides on the Complex Formation of ABC Transporters

Graphical Abstract

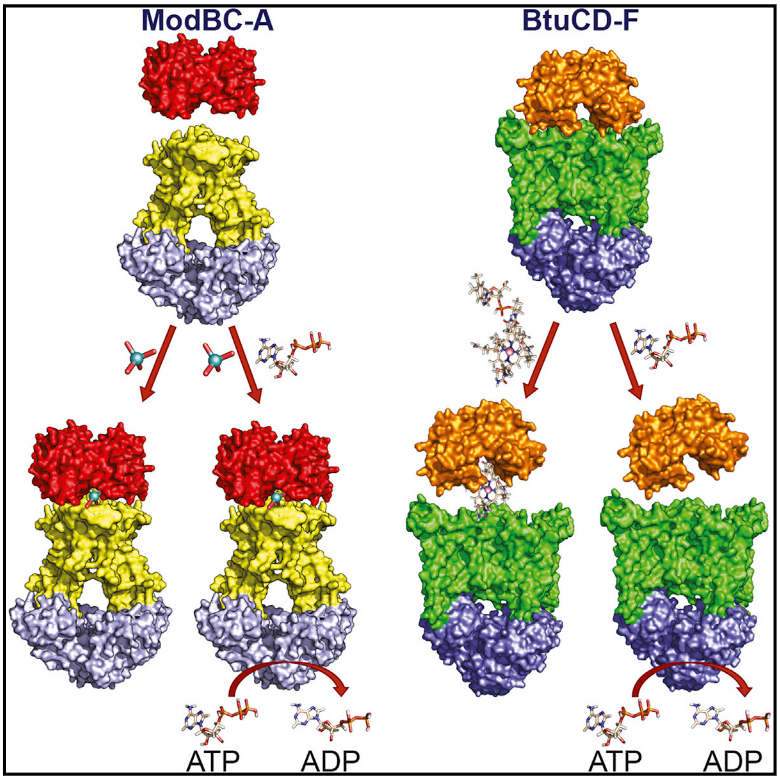

Highlights

- Intact complexes of two ABC transporters are observed using native mass spectrometry
- Substrate and nucleotide binding affects the formation and stability of the complexes
- Molybdate is needed to allow docking of ModA onto the transporter ModBC
- ATP acts synergistically with vitamin B_{12} to destabilize the BtuCD-F complex

Authors

Francesco Fiorentino,
Jani Reddy Bolla, Shahid Mehmood,
Carol V. Robinson

Correspondence

carol.robinson@chem.ox.ac.uk

In Brief

Fiorentino et al. investigated the influence of ligand binding on the complex stability of bacterial ABC transporters using native mass spectrometry. Results show substrate and nucleotide-induced destabilization of the vitamin B_{12} importer BtuCD-F. The insights provided in this work add molecular details to the proposed mechanisms of transport.

Fiorentino et al., 2019, Structure *27*, 651–659
April 2, 2019 © 2019 The Authors. Published by Elsevier Ltd.
https://doi.org/10.1016/j.str.2019.01.010

The Different Effects of Substrates and Nucleotides on the Complex Formation of ABC Transporters

Francesco Fiorentino,[1] Jani Reddy Bolla,[1] Shahid Mehmood,[1,2] and Carol V. Robinson[1,3,*]
[1]Department of Chemistry, University of Oxford, South Parks Road, Oxford OX1 3QZ, UK
[2]Present address: The Francis Crick Institute, 1 Midland Road, London NW1 1ST, UK
[3]Lead Contact
*Correspondence: carol.robinson@chem.ox.ac.uk
https://doi.org/10.1016/j.str.2019.01.010

SUMMARY

The molybdate importer (ModBC-A of *Archaeoglobus fulgidus*) and the vitamin B_{12} importer (BtuCD-F of *Escherichia coli*) are members of the type I and type II ABC importer families. Here we study the influence of substrate and nucleotide binding on complex formation and stability. Using native mass spectrometry we show that the interaction between the periplasmic substrate-binding protein (SBP) ModA and the transporter ModBC is dependent upon binding of molybdate. By contrast, vitamin B_{12} disrupts interactions between the transporter BtuCD and the SBP BtuF. Moreover, while ATP binds cooperatively to BtuCD-F, and acts synergistically with vitamin B_{12} to destabilize the BtuCD-F complex, no effect is observed for ATP binding on the stability of ModBC-A. These observations not only highlight the ability of mass spectrometry to capture these importer-SBP complexes but allow us to add molecular detail to proposed transport mechanisms.

INTRODUCTION

ATP binding cassette (ABC) transporters are a superfamily of membrane proteins that couple the hydrolysis of ATP to the translocation of a diverse range of molecules across lipid bilayers of biological membranes. The basic ABC transporter architecture consists of four core domains: two transmembrane domains (TMDs), and two nucleotide binding domains (NBDs). The two TMDs interact to form a central path, allowing the substrate to be either imported into, or exported out of, the cytoplasm. The NBDs contain ATP binding pockets with ATPase activity: ATP hydrolysis provides the energy required for conformational changes that enable passage of substrates through the TMDs (Rees et al., 2009). ABC exporters are typically homodimers, in which TMDs and NBDs are encoded by a single protein chain (Dawson and Locher, 2006). In contrast, importers have a tetrameric structure with their TMDs and NBDs expressed as separate proteins, present exclusively in internal membranes of prokaryotes and archaea. Whereas NBDs are structurally similar and contain highly conserved motifs, the TMDs of different transporters vary widely in sequence and structure, a feature linked to different mechanisms of transport.

Depending on the fold of the TMDs, ABC importers can be classified as type I, type II, or energy-coupling factor transporters identified recently (Swier et al., 2015; Locher, 2016; Rempel et al., 2018). Type I and type II importers require a periplasmic substrate-binding protein (SBP) for their function. SBPs capture substrates and deliver them to the transporters allowing the passage of the ligand through the lipid bilayer (Maqbool et al., 2015). The molybdate transporter ModBC-A (Hollenstein et al., 2007), along with the maltose transporter MalFGK$_2$ (Chen et al., 2001) and vitamin B_{12} transporter BtuCD-F, are the most studied type I and type II importers, respectively (Locher et al., 2002). In addition to different numbers of TMDs, another feature that distinguishes the two classes of importers is a lack of a substrate binding sites (SBS) in the TMDs of type II importers (Korkhov et al., 2012).

Type I importers mediate the translocation of small molecules such as ions, amino acids, small peptides, and mono- and oligosaccharides, and are characterized by a broad range of binding affinities (Berntsson et al., 2010). Structural and biophysical studies have shown that type I importers employ an alternating access transport model in which conformational changes expose the SBS to alternate faces either side of the membrane (Chen, 2013). In the resting state, the transporter has an inward-facing conformation, which changes to outward-facing once the substrate-loaded binding protein interacts with the periplasmic face. This change of conformation allows substrate release from the SBP, and passage through the TMDs into the SBS. ATP hydrolysis then powers the opening of the NBDs, and the substrate is released into the cytoplasm. Previous studies have indicated that there are subtle differences in the transport mechanism, even within the same class of importers. The type I maltose ABC importer MalFGK$_2$ was shown to bind to its cognate SBP both in the presence and in the absence of maltose, and in both apo and ADP-bound states (Bohm et al., 2013). By contrast, surface plasmon resonance studies indicate that while molybdate is required to initiate the binding of the SBP in *Archaeoglobus fulgidus* ModBC-A, this is not the case for *Haemophilus influenzae* ModBC-A, resulting in substrate-dependent mechanistic differences between the two transporters (Vigonsky et al., 2013).

Type II importers are responsible for the high-affinity uptake of larger molecules, such as heme and vitamin B_{12}, as well as smaller ones, including inorganic ions (Pinkett et al., 2007; Rice et al., 2013), and possess a more complex mechanism of transport than type I importers. The X-ray structure of the entire BtuCD-F complex revealed critical interactions between the

This is an open access article under the CC BY license (http://creativecommons.org/licenses/by/4.0/).

transporter and the SBP and shed light on the post-translocation conformation of the complex (Hvorup et al., 2007). The crystal structures of AMP-PNP-bound BtuCD-F and BtuCD, provide mechanistic details of the ATP-powered release of vitamin B_{12} into the cytoplasm (Korkhov et al., 2012, 2014). These structures suggest that, upon ATP binding, the transmembrane helices undergo a rearrangement to create a cleft, which temporarily is able to accommodate substrate. The absence of substrate-bound co-crystal structures, however, limits detailed knowledge of the transport cycle. Moreover, recent studies provided insights into the effects of ATP binding and hydrolysis on complex formation which led to differing conclusions. Specifically, microscale thermophoresis assays showed that ATP binding and hydrolysis strengthen the interaction between BtuCD and BtuF (Korkhov et al., 2014). By contrast, single-molecule fluorescence imaging revealed that membrane-embedded BtuCD formed a stable complex with BtuF and found that addition of ATP alters the interaction between the two proteins (Goudsmits et al., 2017). Given the fact that the molecular details of the coupled effect of substrate and ATP binding remain unclear (Lewinson et al., 2010; Joseph et al., 2011, 2014) we set out to resolve these effects for these two importers using native mass spectrometry (MS), a technique that enables the analysis of the subunit stoichiometry and ligand binding properties of membrane proteins encapsulated in detergents micelles (Barrera et al., 2009; Gault et al., 2016). Native MS can also be used to report on the population of different complexes in solution by comparing the intensities of ligand-bound charge states within a particular protein system (Patrick et al., 2018; Mehmood et al., 2016b; Bolla et al., 2018; Yen et al., 2018).

The first native MS experiments were developed and applied to BtuCD and revealed that the non-covalent tetramer could be preserved after release from detergent micelles with post-translational modifications and nucleotide binding intact (Barrera et al., 2008). At that time, it was not possible, however, to capture interactions with the substrate binding protein BtuF. To do this, MS conditions needed to be further developed to maintain interactions between transmembrane subunits with both cytoplasmic and periplasmic subunits. To maintain interactions either side of the membrane we optimized further MS conditions and selected ModBC-A from *A. fulgidus* and BtuCD-F from *E. coli* as representatives of type I and type II importer families, respectively (Figure 1A). Following purification of wild-type ModBC, BtuCD, and their respective SBPs, we investigated their lipid binding properties, as well as the effect of substrate, and ATP binding and hydrolysis on complex formation and dissociation of these ABC importers.

RESULTS

Substrates Have Different Effects on the Formation of Type I and Type II Transporter Complexes

We expressed and purified BtuCD following established protocols (see the STAR Methods section) (Locher et al., 2002). We then performed a detergent screen to establish conditions that preserve the complex for native MS (Laganowsky et al., 2013). Selecting detergents from different classes, we found that tetraethylene glycol monooctyl ether (C_8E_4) both solubilized the membrane protein complex and yielded well-resolved charge states corresponding to the mass of intact BtuCD (Figure S1A and Table 1). Adducts were also observed consistent with the presence of co-purified lipids, particularly lipopolysaccharides (LPS), an observation typical of the native mass spectra of membrane proteins (Reading et al., 2015; Bolla et al., 2018) and in particular of ABC transporters (Bechara et al., 2015; Mehmood et al., 2016a).

Having optimized the MS conditions to detect BtuCD, we then purified BtuF (Hvorup et al., 2007) (Figure S3A) and studied its binding properties to BtuCD. To form the BtuCD-F complex, we incubated BtuF with BtuCD and introduced this solution to the mass spectrometer. We observed a new charge state series corresponding to the intact BtuCD-F (>90%) complex (Figure 1B). Associated with the charge state series for the intact complex is a series of satellite peaks indicating that the complex has retained binding to membrane lipids.

For ModBC-A, we expressed and purified ModBC as described previously (Hollenstein et al., 2007) and performed a detergent screen to obtain well-resolved mass spectra. We selected C_8E_4 to replicate the MS conditions used for BtuCD and similarly the experimental mass is consistent with the intact tetramer of ModBC with various associated phospholipids and LPS (Figure S1B). Despite the fact that BtuCD and ModBC were purified using different detergents the same type of bulk lipids from *E. coli* remained bound to the transporters (Figure S2).

To record the mass spectrum of the entire ModBC-A complex we purified ModA (Hollenstein et al., 2007) (Figure S3B) and incubated it with a solution containing ModBC. The most abundant species was ModBC; only a small population had formed a complex with ModA. Moreover, mass spectra also indicated the presence of free ModA in solution, confirming that the complex had not formed (Figure S1C) and implying that additional components are required to achieve full complexation in solution. We reasoned that substrate may be required and added an excess of sodium molybdate to a solution of ModA before buffer exchange and then incubated this solution with ModBC. Under these conditions, we observed a different pattern of charge states whose deconvolution yields a mass consistent with the presence of 100% full ModBC-A complex (Figure 1C). Considering the deconvoluted masses of the individual proteins and the mass of the full complex we calculated a difference of 168 Da, consistent with the presence of molybdate ions bound to the intact complex (calculated as 160 Da). Additional satellite peaks, similar to those observed for ModBC, were assigned to lipid-bound species, associated with the full ModBC-A•MoO_4^{2-} complex.

We suggest that type I importers, exemplified here by ModBC-A, have low affinity for their cognate SBP and only the addition of the substrate triggers the formation of the full complex. By contrast, type II importers, exemplified here by BtuCD-F, bind readily to their cognate SBP, even in the absence of substrate. Taken together, these results imply that the two transporters examined here have different affinities toward their respective SBPs.

Vitamin B_{12} and Cyanocobinamide Destabilize the BtuCD-BtuF Interaction

Having demonstrated that BtuCD and BtuF form a stable complex, the next question was to examine whether vitamin B_{12} had an effect on the stability of the BtuCD-F complex. We

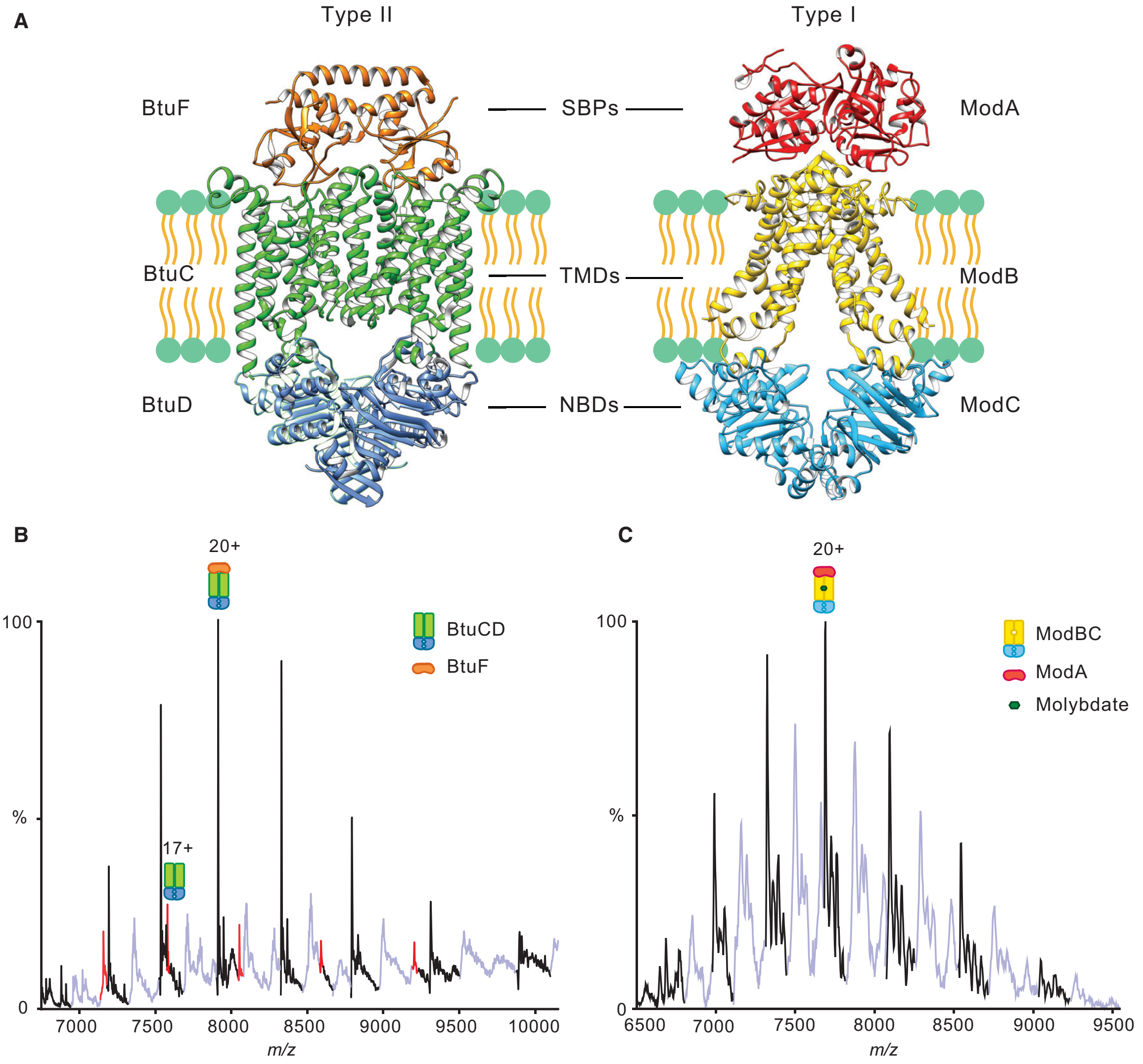

Figure 1. Structures of BtuCD-F and ModBC-A and Their Respective Mass Spectra

(A) X-ray crystal structures of BtuCD-F (left, PDB: 2QI9) and ModBC-A (right, PDB: 2ONK) visualized using PyMol (Schrödinger).

(B) Mass spectrum of BtuCD-F reveals a charge state series consistent with the mass of the tetrameric transporter ($BtuC_2D_2$) bound to the SBP (BtuF) ($BtuC_2D_2$-F) a smaller population of uncomplexed transporter ($BtuC_2D_2$ red peaks) is also apparent. Different lipid species, primarily LPS, are bound to the transporter both in the presence and the absence of the SBP (light blue peaks).

(C) Mass spectrum of ModBC-A shows a charge state series consistent with the tetrameric transporter bound to SBP and molybdate with different lipid-bound species primarily LPS (light blue peaks). Theoretical and observed molecular masses of all species are presented in Table 1.

supplemented BtuF with an excess of vitamin B_{12} and recorded a mass spectrum of the BtuF:vitamin B_{12} complex (Figure 2A, inset). We then incubated the vitamin B_{12}-loaded BtuF with a solution of BtuCD. Interestingly, vitamin B_{12} binding was not detected in either complex (BtuCD or BtuCD-F) (Figure 2A). Absence of vitamin B_{12} is in line with the previous observation that the TMDs of BtuCD lack a high-affinity SBS (Korkhov et al., 2012). The binding of vitamin B_{12} is likely transient, making it difficult to detect with biophysical methods. We noticed, however, an increase in the intensity of charge states corresponding to uncomplexed BtuCD (45%), compared with BtuCD-F in the absence of substrate wherein only 10% of BtuCD was observed (Figures 2A and 1B, red peaks). The presence of vitamin B_{12}, although not detected directly within the complex, destabilizes the interactions between the SBP and the transporter.

Recently, it has been reported that cyanocobinamide, a closely related analog of vitamin B_{12}, is imported by BtuCD-F, and the co-crystal structure of BtuF-cyanocobinamide has been determined (Mireku et al., 2017a). To further investigate this interaction using native MS we added an excess amount of dicyanocobinamide to BtuF alone and observed two series of charge states, corresponding to apo-BtuF and BtuF-cyanocobinamide (Figure 2B, inset), confirming binding to BtuF in line with previous results (Mireku et al., 2017a). We then incubated dicyanocobinamide-loaded BtuF with a solution of BtuCD and observed that the BtuCD and BtuCD-F peak intensities had

Table 1. Theoretical and Observed Masses of all Species Presented in the Current Study

Species	Theoretical Mass (Da)	Observed Mass (Da)	Difference (Da)
BtuCD	128,839	128,837	−2
ModBC	115,011	115,015	+4
BtuF	29,414	29,412	−2
BtuF•Vitamin B_{12}	30,770	30,768	−2
BtuF•Cyanocobinamide	30,430	30,427	−3
ModA	38,584	38,583	−1
BtuCD-F	158,253	158,251	−2
BtuCD-F•2ADP	159,107	159,105	−2
BtuCD-F•2AMP-PNP	159,313	159,320	+7
ModBC-A•MoO_4^{2-}	153,763	153,766	+3
ModBC-A•MoO_4^{2-}•2ADP	154,615	154,623	+8

Theoretical masses were calculated assuming the loss of the first methionine on BtuCD, ModBC, and ModA.

increased to similar values as those observed when vitamin B_{12} was added (Figure 2B), indicating that the complex was destabilized.

The destabilization effect of vitamin B_{12} on the complex was further confirmed by using a molecule with a different structure, vitamin B_9 (folic acid, Figure 2B). Under analogous conditions to those used for vitamin B_{12}, vitamin B_9 did not affect the stability of the complex BtuCD-F with little dissociation of BtuCD (15%) (Figure 2C). The absence of any effects of vitamin B_9 highlights the specificity of the effect for vitamin B_{12} and cyanocobinamide, which, upon binding, induce dissociation of BtuF. We conclude therefore that type II importer BtuCD-F exists in solution as a stable complex with its SBP, even in the absence of ligands. Addition of substrates to this type II importer has a disruptive effect on protein interactions leading to reduced stability of the substrate binding protein.

ATP Binding Acts Synergistically with the Substrate to Decrease BtuCD-BtuF Stability

To gain further insights into the vitamin B_{12} translocation mechanism we studied the effects of ATP binding and turnover on complex stability. We added an excess of ATP/Mg^{2+} to a solution of BtuCD, prior to buffer exchange into MS buffer, and incubated it with a solution of BtuF following the same protocol as above. After a 10-min incubation time the mass spectrum indicates the presence of three BtuCD-F species in solution: the apo form and two nucleotide-bound states with one and two ADP molecules bound, referred to as apo-BtuCD-F, BtuCD-F•1ADP, and BtuCD-F•2ADP, respectively (Figures 3A and S4A). To distinguish the effect of binding from hydrolysis, we carried out analogous experiments in the presence of AMP-PNP, a non-hydrolysable ATP analog. We observed apo-BtuCD-F as well as a population assigned to BtuCD-F•2AMP-PNP (Figures 3B and S4B). Interestingly, while BtuCD-F•2ADP is predominant, apo-BtuCD-F is more abundant than BtuCD-F•1ADP, and in the presence of AMP-PNP no population of a BtuCD-F bound to a single AMP-PNP species was detected. This binding pattern of nucleotides was found to be insensitive to longer incubation periods suggesting that equilibrium had been reached. Overall, our findings suggest that binding of the first nucleotide increases the affinity of the protein for the second one, a characteristic feature of cooperative binding (Cubrilovic et al., 2014). Moreover, in both cases, BtuCD was bound to the two different nucleotides in a similar fashion to that observed for the full complex. The overall population of free BtuCD and corresponding adducts was found to increase after addition of nucleotides, with the BtuCD/BtuCD-F ratio being in the range of 40%–45% in the presence of ATP or AMP-PNP (Figures 3A, 3B, S4A, and S4B). Such an increase in BtuCD-related charge states is analogous to the situation observed for vitamin B_{12}, thus indicating that a destabilizing effect is also induced by nucleotide binding.

To study the combined effect of substrate and nucleotide, we added solutions of ATP and vitamin B_{12} simultaneously to the BtuCD-F complex. Interestingly, we noticed that the increase in the intensity of free BtuCD charge states was even more significant when both ligands are present (Figures S4A, S4C, and 1B), implying a synergistic effect of binding both ATP and vitamin B_{12}. Our data also show that the cooperativity in ATP binding observed above is retained in the presence of vitamin B_{12} (Figure S4C). The results obtained from the addition of ATP, AMP-PNP, and ATP-vitamin B_{12} suggest a mechanism whereby ATP binding causes a conformational change that leads to a decrease in affinity for the substrate binding protein. While the AMP-PNP experiments cannot exclude a contribution from ATP hydrolysis, in destabilizing the complex *in vivo*, they do demonstrate that nucleotide binding alone is sufficient to destabilize the interaction between BtuCD and BtuF.

ATP Binding Does Not Alter ModBC-ModA Interactions

To evaluate the impact of ATP binding and hydrolysis on the ModBC-A complex we first added an excess of ATP to a solution of ModBC before exchanging into an MS-compatible buffer. We then incubated this solution with ModA. The resulting mass spectrum did not display any nucleotide binding, implying that all ATP had been removed during buffer exchange. To observe nucleotide binding, we added excess ATP to ModA pre-loaded with sodium molybdate and recorded the mass spectrum under optimized conditions. Three populations of ModBC-A•MoO_4^{2-} are observed: an apo form and the species bound to one and two ADP molecules (Figures 3C and S5). The distribution of the three species is different from BtuCD-F; indeed, the apo form is less abundant, thus no cooperativity is observed in ATP/ADP binding to ModBC-A•MoO_4^{2-}, indicating little or no cooperativity of the two ATP binding events. Moreover, we did not observe additional charge states corresponding to ModBC, implying that ATP did not influence the ModBC-A/ModBC interactions significantly.

Based on our results we propose that nucleotide binding has a different impact on type I and type II transporters examined here. In the type I transporter, ATP did not perturb interactions between the transporter and its cognate SBP. Nucleotide binding was found to be independent of the effect of substrate, the latter being pivotal for complex formation. By contrast, ATP binding and hydrolysis modulates interactions between the transporter and the SBP in the transport cycle of the type II importer. In addition, ATP binding follows different patterns in the two complexes: while we observed cooperative ATP binding in the case of

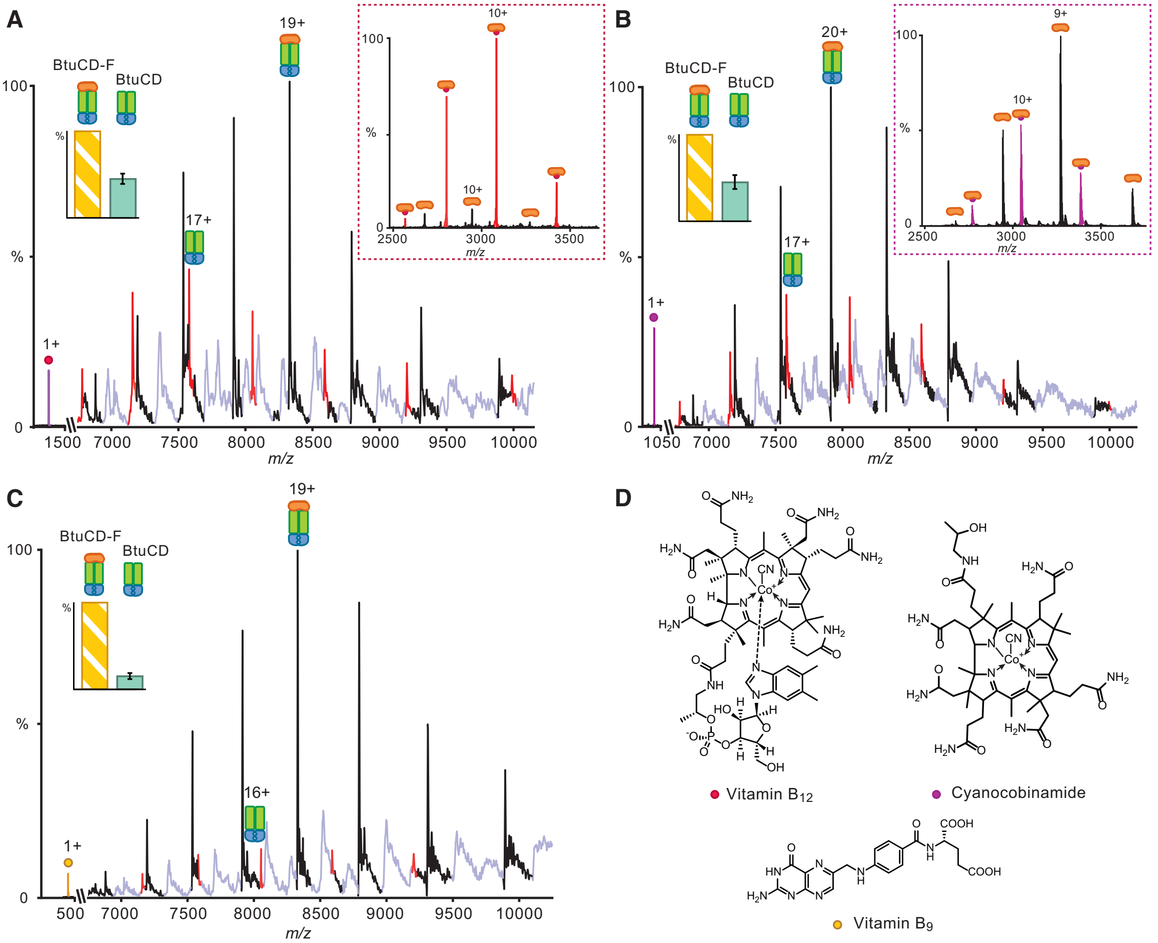

Figure 2. BtuCD-F Is Destabilized by Vitamin B_{12} and Cyanocobinamide

(A) Mass spectrum of BtuCD-F recorded after addition of 2 mM vitamin B_{12} to BtuF before buffer exchange. An increase in the intensity of the charge states corresponding to uncomplexed BtuCD (red peaks 45% with respect to BtuCD-F 100%) is observed in the presence of vitamin B_{12} (purple peak) compared with in the absence of ligand (cf. Figure 1B). The inset shows the mass spectrum of BtuF-vitamin B_{12} complex after addition of 2 mM vitamin B_{12}.

(B) Mass spectrum of BtuCD-F after addition of 2 mM dicyanocobinamide to BtuF before buffer exchange. The ratio of BtuCD (red peaks 44% with respect to BtuCD-F 100%) is closely similar to the extent of complex formation in the presence of vitamin B_{12}. The inset shows the mass spectrum of BtuF-cyanocobinamide complex after addition of 2mM dicyanocobinamide.

(C) Mass spectrum of BtuCD-F after addition of 2 mM vitamin B_9 to BtuF before buffer exchange. The ratio of BtuCD (red peaks 15% with respect to BtuCD-F 100%) in the presence of vitamin B_9 is closely similar to the extent of complex formation in the absence of ligand. Data are represented as means ± SD (n = 3).

(D) Chemical structures of the molecules used for the binding experiments.

BtuCD-F, we did not observe cooperativity for ModBC-A. While we cannot rule out cooperativity of nucleotide binding for type I importers we speculate that differences in the TMD structures of the two transporters may influence interactions and crosstalk within the NBDs.

DISCUSSION

In the current study, we employed native MS to observe membrane protein complexes developing the approach further to capture ABC importers bound to their cognate substrate binding proteins. Capturing these complexes allowed us to explore the influence of ligands on their stability. Specifically, we showed that the two ABC importers were associated with similar lipids even when extracted and purified in different detergents. Moreover, the cohort of bound lipids did not change significantly when binding of ATP or substrates or during complex formation. These observations suggest that the transporters do not have a specific preference for the bulk lipids that surround them, in contrast to the flippase TmrAB investigated previously, wherein negatively charged lipids bound tightly to the protein (Bechara et al., 2015). We also demonstrated that binding of BtuCD-F substrates, vitamin B_{12} and cyanocobinamide, led to disruption of the BtuCD-BtuF complex, whereas molybdate binding is required to promote docking of ModA onto ModBC. The dissociation of BtuCD-F complex is explained by the fact that, once vitamin B_{12}-loaded BtuF docks on to BtuCD, insufficient space is available to accommodate the bulky B_{12} substrate (Figure 2B). In addition, the ModBC-A complex is stable, regardless of the presence of ATP, while BtuCD-F is destabilized by binding of

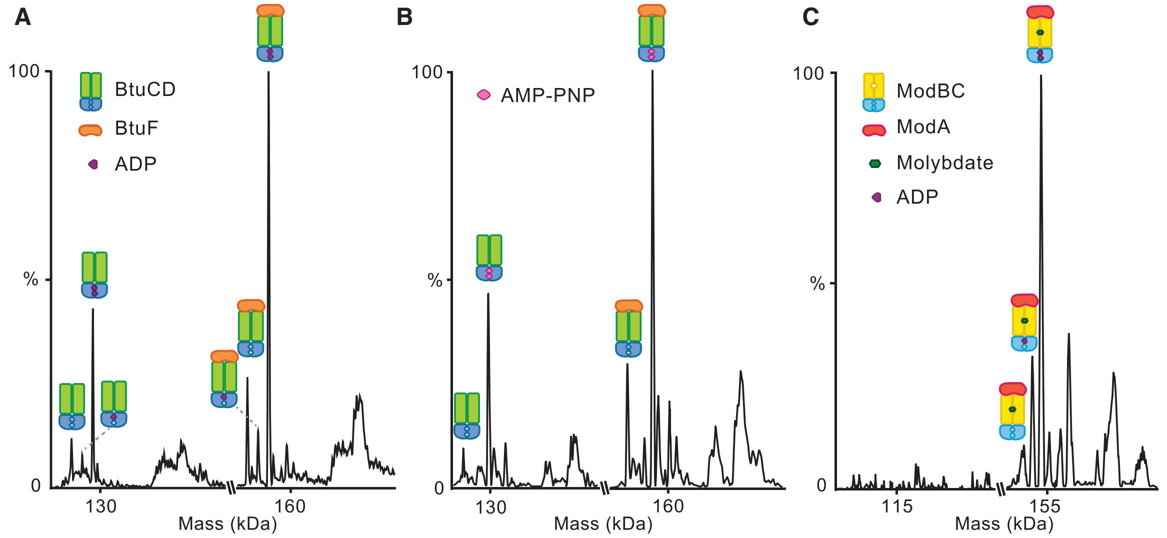

Figure 3. Effect of Nucleotides on BtuCD-F and ModBC-A Complex Formation

(A) Deconvoluted masses of the spectrum of BtuCD-F recorded after addition of 5 mM ATP to BtuCD before buffer exchange. The presence of ADP-bound species of both BtuCD-F and BtuCD are observed with similar ratios. The relative abundance of BtuCD in this spectrum is 40% of the BtuCD-F signal.

(B) Deconvoluted masses of the spectrum of BtuCD-F recorded after addition of 5 mM AMP-PNP to BtuCD before buffer exchange. Also in this case, AMP-PNP-bound species are detected. The relative abundance of BtuCD in this spectrum is 43% of the BtuCD-F signal.

(C) Deconvoluted masses of the spectrum of ModBC-A recorded following the addition of 10 μM ATP to ModBC after buffer exchange. ModBC-A species bound to zero, one and two ADP molecules are observed and no ModBC alone is detected.

ATP or its non-hydrolysable analog AMP-PNP. Moreover, the disruptive effect of vitamin B_{12} is enhanced by the presence of nucleotides, consistent with the ligands acting synergistically to promote substrate release. We also highlighted further differences in ATP binding mechanisms between type I and type II transporters. While no significant cooperativity was detected for ModBC-A, when binding to nucleotides, for BtuCD-F the first ATP binding event facilitates the second in accordance with typical cooperative binding models.

On the basis of our results, and with contributions from previous structural and biophysical data (Hvorup et al., 2007; Vigonsky et al., 2013; Lewinson et al., 2010; Joseph et al., 2011, 2014; Korkhov et al., 2012, 2014; Goudsmits et al., 2017), we present two different transport models for type I and type II ABC importers.

For the type I importer ModBC-A our data show that in the absence of substrate the extent of SBP binding onto the transporter is very low; essentially, there is no complex formation (Figure 4A, state I). Binding of substrate results in increased complex formation, triggering the docking of the SBP onto the transporter, trapping the substrate and beginning of the transport cycle (Figure 4A, state II). This interaction is then thought to initiate a conformational rearrangement, converting the TMDs from an inward- to an outward-facing conformation (Oldham and Chen, 2011), and likely forming a tunnel which allows the translocation of the substrate from the SBP to the SBS, located in the TMDs (Figures 4A, state III and 1B). Subsequent ATP binding induces a further rearrangement to an inward-facing conformation causing dissociation of the substrate from the SBS and its release into the cytoplasm (Figure 4A, state IV). The translocation of the substrate and dissociation of ADP from the NBDs reset the transporter to the initial state (Figure 4A, state I). Together these steps are consistent with the alternating access transport mechanism in which conformational changes expose the SBS to alternate faces either side of the membrane (Chen, 2013; Khare et al., 2009).

The type II importer (BtuCD-F) studied here follows a different cycle wherein the transporter complex with the SBP is stable in solution, even in the absence of substrate (Figures 4B state I and 1B). Following the cooperative binding of ATP, the TMDs undergo conformational changes leading to a disruption of the interactions with the SBP. The reduction in the level of complexation does not necessarily imply that appreciable dissociation takes place *in vivo*, rather it suggests that the complex is undergoing conformational changes whereby binding of SBP to the TMDs is perturbed. We propose a model whereby the SBP-bound transporter partially opens upon nucleotide binding, consistent with the increased levels of uncomplexed transporter observed by MS (Figure 4B state II) (Goudsmits et al., 2017). Moreover, earlier studies showed that type II importers are characterized by a high level of futile ATP hydrolysis, with a high ATP:vitamin B_{12} transport ratio (Borths et al., 2005; Tal et al., 2013). Once the complex opens, substrate is bound (Figure 4B, state III) and partially accommodated in a cleft formed by the helices present in the TMDs (Korkhov et al., 2014) as a consequence of nucleotide binding. However, the presence of substrate and the full occupancy of nucleotide binding sites has a synergistic effect rendering the full complex more susceptible to dissociation, leading to the dissociation of ADP and Pi, which triggers the subsequent translocation of the substrate into the cytoplasm (Figure 4B, state IV). The presence of substrate decreases the stability of the complex; therefore, a critical factor in the release of the substrate to the cytoplasm is the achievement of the state of minimal energy represented by the SBP-bound transporter (Figure 4B state I).

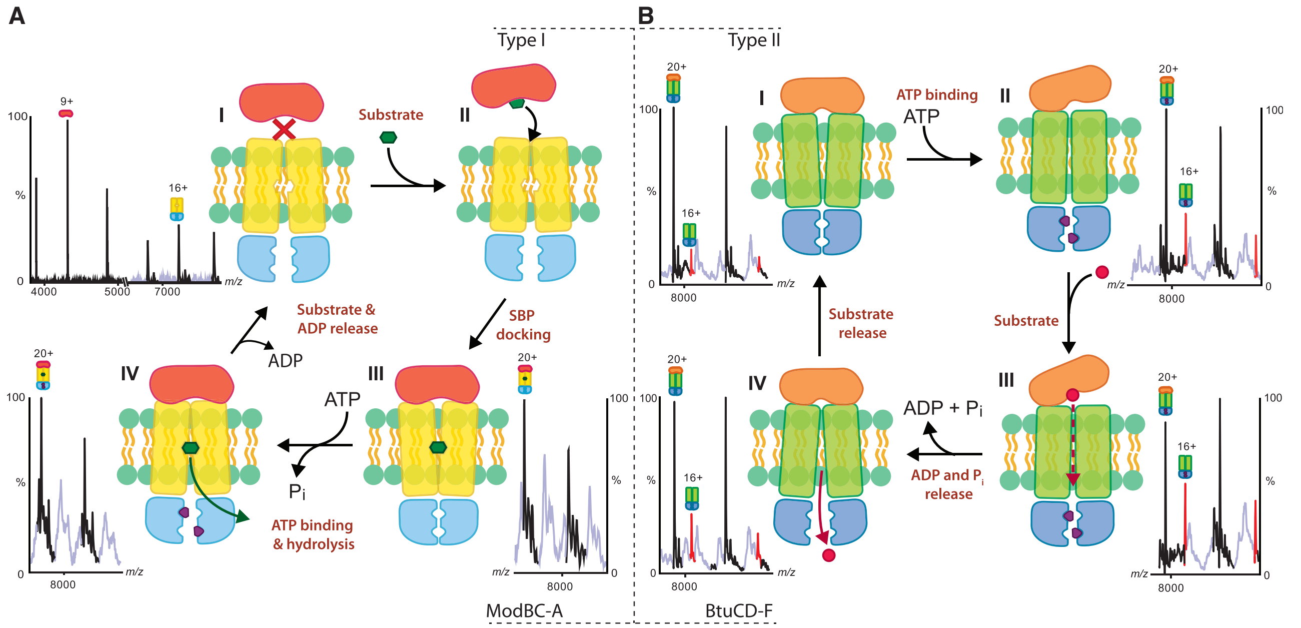

Figure 4. Transport Models for Type I Importer ModBC-A and TYPE II IMPorter BtuCD-F

(A) Transport model for ModBC-A. The formation of the complex in the absence of molybdate is unfavorable (state I). Binding of molybdate triggers the docking of ModA onto ModBC (state II). Following a rearrangement of the TMDs, molybdate moves toward the SBS (state III). ATP binding and hydrolysis triggers a further conformational change that translocate the substrate into the cytoplasm (state IV). The release of ADP resets the transporter to the resting state (state I).

(B) Transport model for BtuCD-F. BtuCD and BtuF form a stable complex even in the absence of vitamin B_{12} (state I). ATP binds cooperatively, leading to a partial displacement of BtuF from BtuCD (state II). When vitamin B_{12} is available, a transient BtuCD-F•2ADP•B_{12} complex is formed (state III). This unstable conformation is relaxed through ADP and Pi dissociation, followed by vitamin B_{12} release (state IV) leading to the resetting of the transporter.

Over the past years, biochemical techniques such as surface plasmon resonance (Vigonsky et al., 2013; Qasem-Abdullah et al., 2017) and single-molecule fluorescence resonance energy transfer (Yang et al., 2018; Husada et al., 2018) have been used to study the binding of ligands on ABC transporters, providing valuable information about their influence on the mechanism of transport. The MS approach we have applied here, therefore, complements earlier studies (Lewinson et al., 2010; Korkhov et al., 2014; Goudsmits et al., 2017) by providing evidence of the role of substrate and ATP in complex formation and substrate transport across the lipid bilayer. These results further indicate that there is an essential difference between type I and type II ABC importers examined here. Moreover, ABC transporters have a pivotal role in bacterial life and therefore represent optimal targets for the development of antimicrobial drugs (Mireku et al., 2017b). Given the increasing number of multidrug-resistant bacteria, there is an urgent need for new strategies targeting less explored pathways. Hence, better understanding of ABC importers mechanisms may prove useful for the development of novel therapeutics.

STAR★METHODS

Detailed methods are provided in the online version of this paper and include the following:

- KEY RESOURCES TABLE
- CONTACT FOR REAGENT AND RESOURCE SHARING
- METHOD DETAILS
  - Plasmid Preparation and Protein Expression
  - Purification of Soluble Proteins
  - Purification of Membrane Proteins
  - Native MS Experiments
  - Lipidomics Analysis
- QUANTIFICATION AND STATISTICAL ANALYSIS

SUPPLEMENTAL INFORMATION

Supplemental Information includes five figures and can be found with this article online at https://doi.org/10.1016/j.str.2019.01.010.

ACKNOWLEDGMENTS

The research in the Robinson laboratory is supported by an MRC program grant (MR/N020413/1), an ERC Advanced Grant ENABLE (695511) and a Wellcome Trust Investigator Award (104633/Z/14/Z). F.F. holds a SABS CDT Studentship supported by the EPSRC and the MRC (EP/L016044/1). BtuCD and ModBC plasmids were a kind gift from Kaspar Locher (ETH Zurich). A. Howes and D. Wu (University of Oxford) are acknowledged for proofreading the manuscript and help in lipidomics analysis, respectively.

AUTHOR CONTRIBUTIONS

F.F., J.R.B., S.M., and C.V.R. designed the study. F.F. and J.R.B. prepared BtuF and ModA constructs. F.F. expressed and purified all proteins and performed all MS measurements. F.F. analyzed the data with the help of J.R.B. and S.M. F.F. and C.V.R. wrote the paper with support from J.R.B and S.M.

DECLARATION OF INTEREST

C.V.R. is a co-founder and consultant of OMass Therapeutics.

Received: August 8, 2018
Revised: November 17, 2018
Accepted: January 18, 2019
Published: February 21, 2019

REFERENCES

Barrera, N.P., Di Bartolo, N., Booth, P.J., and Robinson, C.V. (2008). Micelles protect membrane complexes from solution to vacuum. Science *321*, 243–246.

Barrera, N.P., Isaacson, S.C., Zhou, M., Bavro, V.N., Welch, A., Schaedler, T.A., Seeger, M.A., Miguel, R.N., Korkhov, V.M., van Veen, H.W., et al. (2009). Mass spectrometry of membrane transporters reveals subunit stoichiometry and interactions. Nat. Methods. *6*, 585–587.

Bechara, C., Noll, A., Morgner, N., Degiacomi, M.T., Tampe, R., and Robinson, C.V. (2015). A subset of annular lipids is linked to the flippase activity of an ABC transporter. Nat. Chem. *7*, 255–262.

Berntsson, R.P., Smits, S.H., Schmitt, L., Slotboom, D.J., and Poolman, B. (2010). A structural classification of substrate-binding proteins. FEBS Lett. *12*, 2606–2617.

Bohm, S., Licht, A., Wuttge, S., Schneider, E., and Bordignon, E. (2013). Conformational plasticity of the type I maltose ABC importer. Proc. Natl. Acad. Sci. U S A *110*, 5492–5497.

Bolla, J.R., Sauer, J.B., Wu, D., Mehmood, S., Allison, T.M., and Robinson, C.V. (2018). Direct observation of the influence of cardiolipin and antibiotics on lipid II binding to MurJ. Nat. Chem. *10*, 363–371.

Borths, E.L., Poolman, B., Hvorup, R.N., Locher, K.P., and Rees, D.C. (2005). In vitro functional characterization of BtuCD-F, the *Escherichia coli* ABC transporter for vitamin B12 uptake. Biochemistry *44*, 16301–16309.

Chen, J. (2013). Molecular mechanism of the *Escherichia coli* maltose transporter. Curr. Opin. Struct. Biol. *23*, 492–498.

Chen, J., Sharma, S., Quiocho, F.A., and Davidson, A.L. (2001). Trapping the transition state of an ATP-binding cassette transporter: evidence for a concerted mechanism of maltose transport. Proc. Natl. Acad. Sci. U S A *98*, 1525–1530.

Cubrilovic, D., Haap, W., Barylyuk, K., Ruf, A., Badertscher, M., Gubler, M., Tetaz, T., Joseph, C., Benz, J., and Zenobi, R. (2014). Determination of protein-ligand binding constants of a cooperatively regulated tetrameric enzyme using electrospray mass spectrometry. ACS Chem. Biol. *9*, 218–226.

Dawson, R.J.P., and Locher, K.P. (2006). Structure of a bacterial multidrug ABC transporter. Nature *443*, 180–185.

Gault, J., Donlan, J.A., Liko, I., Hopper, J.T., Gupta, K., Housden, N.G., Struwe, W.B., Marty, M.T., Mize, T., Bechara, C., et al. (2016). High-resolution mass spectrometry of small molecules bound to membrane proteins. Nat. Methods *13*, 333–336.

Goudsmits, J.M.H., Slotboom, D.J., and van Oijen, A.M. (2017). Single-molecule visualization of conformational changes and substrate transport in the vitamin B12 ABC importer BtuCD-F. Nat. Commun. *8*, 1652.

Hernández, H., and Robinson, C.V. (2007). Determining the stoichiometry and interactions of macromolecular assemblies from mass spectrometry. Nat. Protoc. *2*, 715–726.

Hollenstein, K., Frei, D.C., and Locher, K.P. (2007). Structure of an ABC transporter in complex with its binding protein. Nature *446*, 213–216.

Husada, F., Bountra, K., Tassis, K., de Boer, M., Romano, M., Rebuffat, S., Beis, K., and Cordes, T. (2018). Conformational dynamics of the ABC transporter McjD seen by single-molecule FRET. EMBO J. *37*, e100056.

Hvorup, R.N., Goetz, B.A., Niederer, M., Hollenstein, K., Perozo, E., and Locher, K.P. (2007). Asymmetry in the structure of the ABC transporter-binding protein complex BtuCD-BtuF. Science *317*, 1387–1390.

Joseph, B., Jeschke, G., Goetz, B.A., Locher, K.P., and Bordignon, E. (2011). Transmembrane gate movements in the type II ATP-binding cassette (ABC) importer BtuCD-F during nucleotide cycle. J. Biol. Chem. *286*, 41008–41017.

Joseph, B., Korkhov, V.M., Yulikov, M., Jeschke, G., and Bordignon, E. (2014). Conformational cycle of the vitamin B12 ABC importer in liposomes detected by double electron-electron resonance (DEER). J. Biol. Chem. *289*, 3176–3185.

Khare, D., Oldham, M.L., Orelle, C., Davidson, A.L., and Chen, J. (2009). Alternating access in maltose transporter mediated by rigid-body rotations. Mol. Cell *33*, 528–536.

Korkhov, V.M., Mireku, S.A., and Locher, K.P. (2012). Structure of AMP-PNP-bound vitamin B12 transporter BtuCD-F. Nature *490*, 367–372.

Korkhov, V.M., Mireku, S.A., Veprintsev, D.B., and Locher, K.P. (2014). Structure of AMP-PNP-bound BtuCD and mechanism of ATP-powered vitamin B12 transport by BtuCD-F. Nat. Struct. Mol. Biol. *21*, 1097–1099.

Laganowsky, A., Reading, E., Hopper, J.T., and Robinson, C.V. (2013). Mass spectrometry of intact membrane protein complexes. Nat. Protoc. *8*, 639–651.

Lewinson, O., Lee, A.T., Locher, K.P., and Rees, D.C. (2010). A distinct mechanism for the ABC transporter BtuCD-BtuF revealed by the dynamics of complex formation. Nat. Struct. Mol. Biol. *17*, 332–338.

Locher, K.P. (2016). Mechanistic diversity in ATP-binding cassette (ABC) transporters. Nat. Struct. Mol. Biol. *23*, 487–493.

Locher, K.P., Lee, A.T., and Rees, D.C. (2002). The *E. coli* BtuCD structure: a framework for ABC transporter architecture and mechanism. Science *296*, 1091–1098.

Maqbool, A., Horler, R.S., Muller, A., Wilkinson, A.J., Wilson, K.S., and Thomas, G.H. (2015). The substrate-binding protein in bacterial ABC transporters: dissecting roles in the evolution of substrate specificity. Biochem. Soc. Trans. *43*, 1011–1017.

Marty, M.T., Baldwin, A.J., Marklund, E.G., Hochberg, G.K., Benesch, J.L., and Robinson, C.V. (2015). Bayesian deconvolution of mass and ion mobility spectra: from binary interactions to polydisperse ensembles. Anal. Chem. *87*, 4370–4376.

Mehmood, S., Corradi, V., Choudhury, H.G., Hussain, R., Becker, P., Axford, D., Zirah, S., Rebuffat, S., Tieleman, D.P., Robinson, C.V., et al. (2016a). Structural and functional basis for lipid synergy on the activity of the antibacterial peptide ABC transporter McjD. J. Biol. Chem. *291*, 21656–21668.

Mehmood, S., Marcoux, J., Gault, J., Quigley, A., Michaelis, S., Young, S.G., Carpenter, E.P., and Robinson, C.V. (2016b). Mass spectrometry captures off-target drug binding and provides mechanistic insights into the human metalloprotease ZMPSTE24. Nat. Chem. *8*, 1152–1158.

Mireku, S.A., Ruetz, M., Zhou, T., Korkhov, V.M., and Locher, K.P. (2017a). Conformational change of a tryptophan residue in BtuF facilitates binding and transport of cobinamide by the vitamin B12 transporter BtuCD-F. Sci. Rep. *7*, 41575.

Mireku, S.A., Sauer, M.M., Glockshuber, R., and Locher, K.P. (2017b). Structural basis of nanobody-mediated blocking of BtuF, the cognate substrate-binding protein of the *Escherichia coli* vitamin B12 transporter BtuCD. Sci. Rep. *7*, 14296.

Oldham, M.L., and Chen, J. (2011). Crystal structure of the maltose transporter in a pretranslocation intermediate state. Science *332*, 1202–1205.

Patrick, J.W., Boone, C.D., Liu, W., Conover, G.M., Liu, Y., Cong, X., and Laganowsky, A. (2018). Allostery revealed within lipid binding events to membrane proteins. Proc. Natl. Acad. Sci. U S A *115*, 2976–2981.

Pinkett, H.W., Lee, A.T., Lum, P., Locher, K.P., and Rees, D.C. (2007). An inward-facing conformation of a putative metal-chelate-type ABC transporter. Science *315*, 373–377.

Qasem-Abdullah, H., Perach, M., Livnat-Levanon, N., and Lewinson, O. (2017). ATP binding and hydrolysis disrupts the high-affinity interaction between the heme ABC transporter HmuUV and its cognate substrate binding protein. J. Biol. Chem. *292*, 14617–14624.

Reading, E., Walton, T.A., Liko, I., Marty, M.T., Laganowsky, A., Rees, D.C., and Robinson, C.V. (2015). The effect of detergent, temperature, and lipid on the oligomeric state of MscL constructs: insights from mass spectrometry. Chem. Biol. *22*, 593–603.

Rees, D.C., Johnson, E., and Lewinson, O. (2009). ABC transporters: the power to change. Nat. Rev. Mol. Cell Biol. *10*, 218–227.

Rempel, S., Stanek, W.K., and Slotboom, D.J. (2018). Energy-coupling factor-type ATP-binding cassette transporters. Annu. Rev. Biochem. *88*, https://doi.org/10.1146/annurev-biochem-013118-111705.

Rice, A.J., Alvarez, F.J., Schultz, K.M., Klug, C.S., Davidson, A.L., and Pinkett, H.W. (2013). EPR spectroscopy of MolB2C2-a reveals mechanism of transport for a bacterial type II molybdate importer. J. Biol. Chem. *288*, 21228–21235.

Swier, L.J.Y.M., Slotboom, D., and Poolman, B. (2015). ABC importers. In ABC Transporters - 40 Years on, A.M. George, ed. (Springer Verlag), pp. 3–36.

Tal, N., Ovcharenko, E., and Lewinson, O. (2013). A single intact ATPase site of the ABC transporter BtuCD drives 5% transport activity yet supports full in vivo vitamin B12 utilization. Proc. Natl. Acad. Sci. U S A *110*, 5434–5439.

Vigonsky, E., Ovcharenko, E., and Lewinson, O. (2013). Two molybdate/tungstate ABC transporters that interact very differently with their substrate binding proteins. Proc. Natl. Acad. Sci. U S A *110*, 5440–5445.

Yang, M., Livnat-Levanon, N., Acar, B., Aykac-Fas, B., Masrati, G., Rose, J., Ben-Tal, N., Haliloglu, T., Zhao, Y., and Lewinson, O. (2018). Single-molecule probing of the conformational homogeneity of the ABC transporter BtuCD. Nat. Chem. Biol. *14*, 715–722.

Yen, H.Y., Hoi, K.K., Liko, I., Hedger, G., Horrell, W., Song, M.R., Wu, D., Heine, P., Warne, T., Lee, Y., et al. (2018). PtdIns(4,5)P2 stabilizes active states of GPCRs and enhances selectivity of G-protein coupling. Nature *559*, 423–427.

STAR★METHODS

KEY RESOURCES TABLE

REAGENT or RESOURCE	SOURCE	IDENTIFIER
Bacterial and Virus Strains		
BL21 (DE3)	New England Biolabs	Cat# C2527I
Stellar Competent Cells	Takara	Cat# 636763
Chemicals, Peptides, and Recombinant Proteins		
In-Fusion cloning kit	Clonetech	Cat # 638909
n-Dodecyl-β-D-Maltopyranoside (DDM)	Anatrace	Cat# D310S
Octaethylene Glycol Monododecyl Ether (C12E8)	Anatrace	Cat# O330
n-Dodecyl-N,N-Dimethylamine-N-Oxide (LDAO)	Anatrace	Cat# D360
Tetraethylene Glycol Monooctyl Ether (C8E4)	Anatrace	Cat# T350
Ammonium acetate	Sigma Aldrich	Cat# A2706
Vitamin B12	Sigma Aldrich	Cat# V2876
Dicyanocobinamide	Sigma Aldrich	Cat# C3021
Folic Acid (Vitamin B9)	Sigma Aldrich	Cat# F7876
Adenosine 5'-triphosphate (ATP)	Sigma Aldrich	Cat# A26209
Magnesium chloride	Sigma Aldrich	Cat# M8266
Adenosine 5'-(β,γ-imido)triphosphate (AMP-PNP)	Sigma Aldrich	Cat# A2647
Sodium molybdate	Sigma Aldrich	Cat# 737860
Deposited Data		
Crystal structure of *A. fulgidus* ModBC-A	Hollenstein et al., 2007	PDB: 2ONK https://www.rcsb.org/structure/2ONK
Crystal structure of *E. coli* BtuCD-F	Hvorup et al., 2007	PDB: 2QI9 https://www.rcsb.org/structure/2QI9
Recombinant DNA		
pET19b-ModBC	Gift from Kaspar Locher	N/A
pET19b-BtuCD	Gift from Kaspar Locher	N/A
ModA (aa 32-342) gene fragment	This paper	N/A
BtuF (aa 23-266) gene fragment	This paper	N/A
pET28a	Novagen	Cat# 69864-3
pET22b	Novagen	Cat# 69744-3
Software and Algorithms		
UniDec	Marty et al., 2015	http://www.unidec.chem.ox.ac.uk
PyMol	Schrödinger, LLC Version 1.8	www.sourceforge.net/projects/pymol
Xcalibur	Thermo Scientific	N/A
Other		
Clark Borosilicate Standard Wall Capillaries	Harvard Apparatus	Cat #30-0044
PicoTip Emitter SilicaTip	New Objective	Cat #FS360-20-10

CONTACT FOR REAGENT AND RESOURCE SHARING

Further information and requests for resources and reagents should be directed to and will be fulfilled by Carol V. Robinson (carol.robinson@chem.ox.ac.uk).

METHOD DETAILS

Plasmid Preparation and Protein Expression

ModA plasmid was obtained by inserting the gene fragment encoding the predicted mature part of the periplasmic binding-protein (amino acids 32-342) into a modified pET-28a expression vector (Novagen) between BamHI and XhoI cloning sites, using an

In-Fusion cloning kit (Clonetech). The modified pET-28a contained an N-terminal decahistidine affinity tag followed by a TEV protease cleavage site. The BtuF plasmid was constructed by inserting the gene fragment containing amino acids 23-266 into pET-22b expression vector (Novagen) between NcoI and XhoI cloning sites. The plasmids used for over-expression of *E. coli* BtuCD and *A. fulgidus* ModBC were a kind gift from Kaspar Locher (ETH Zurich). All plasmids were amplified by transforming them into *E. coli* Stellar Competent Cells (Takara) and the DNA sequences were verified by Sanger sequencing.

For each protein, the plasmid was transformed in *E. coli* BL21(DE3) (New England Biolabs). Several colonies were inoculated into 100 ml LB media and grown overnight at 37°C. One litre of LB in 2 litre shaker flasks was inoculated with 7 ml of overnight culture and grown at 37°C until the culture reached OD600 nm (OD600) between 0.6 and 0.8. Isopropyl-β-D-1-thiogalactopyranoside (IPTG) was added to the culture at a final concentration of 0.5 mM and grown for 3 h at 37°C. Cells were collected by centrifugation at 5,000xg for 10 min at 4°C. Cell pellets were resuspended in buffer containing 150mM NaCl, 20 mM Tris (pH 7.5) and stored at −80°C.

Purification of Soluble Proteins

ModA

Resuspended cells were thawed and supplemented with an EDTA-free protease inhibitor cocktail (Roche). The cell suspension was passed several times through an M-110 PS microfluidizer (Microfluidics) at 15,000 psi. Insoluble material was pelleted by centrifugation at 20,000xg for 20 min at 4°C. The supernatant was filtered before loading onto a 5 ml HisTrap-HP column (GE Healthcare, Piscataway, NJ) equilibrated in 200 mM NaCl, 20 mM Hepes (pH 7.5), 10% glycerol and 20 mM imidazole. After the clarified supernatant was loaded, the column was initially washed with 50 ml of 200 mM NaCl, 20 mM Hepes (pH 7.5), 10% glycerol and 20 mM imidazole, and washed again with 50 ml of 200 mM NaCl, 20 mM Hepes (pH 7.5), 10% glycerol and 80 mM imidazole. The bound protein was eluted with 200 mM NaCl, 20 mM Hepes (pH 7.5), 10% glycerol and 500 mM imidazole. Peak fractions were pooled, incubated with TEV protease and dialysed against 200 mM NaCl, 20 mM Hepes (pH 7.5), and 10% glycerol. The protein was then concentrated and loaded onto the Superdex 200 size exclusion chromatography (SEC) column in 200 mM NaCl, 20 mM Hepes (pH 7.5), and 10% glycerol.

BtuF

The protein was purified following the same protocol as ModA, except that the final buffer contains also 10 mM DTT.

Purification of Membrane Proteins

Resuspended cells were thawed and supplemented with EDTA-free protease inhibitor cocktail (Roche). The cell suspension was passed several times through an M-110 PS microfluidizer (Microfluidics) at 15,000 psi. Insoluble material was pelleted by centrifugation at 20,000xg for 20 min at 4°C. The supernatant was ultracentrifuged at (200,000xg for 1h), and the membrane fractions were collected. Membranes were resuspended in ice-cold buffer containing 150 mM NaCl, 20 mM Tris (pH 7.5) and 20% glycerol. Resuspended membranes were used either directly or flash frozen in liquid nitrogen and stored at −80°C.

ModBC

The protein was solubilized from the membrane fraction with 150 mM NaCl, 20 mM Tris (pH 7.5), 20% glycerol, 1% n-Dodecyl-β-D-Maltopyranoside (DDM, Anatrace) and 1% octaethylene glycol monododecyl ether ($C_{12}E_8$, Anatrace) for 1h at 4°C. Extracted membrane proteins were clarified by centrifugation at 20,000xg for 20 min at 4°C. Supernatant was filtered before loading onto a 5 ml HisTrap-HP column (GE Healthcare, Piscataway, NJ) equilibrated in 150 mM NaCl, 20 mM Tris (pH 7.5), 10% glycerol, 20 mM imidazole and 0.01% $C_{12}E_8$. After the clarified supernatant was loaded, the column was initially washed with 50 ml of 150 mM NaCl, 20 mM Tris (pH 7.5), 10% glycerol, 20 mM imidazole and 0.01% $C_{12}E_8$, and washed again with 50 ml of 150 mM NaCl, 20 mM Tris (pH 7.5), 10% glycerol, 80 mM imidazole and 0.01% $C_{12}E_8$. The bound protein was eluted with 150 mM NaCl, 20 mM Tris (pH 7.5), 10% glycerol, 500 mM imidazole and 0.01% $C_{12}E_8$. The protein was concentrated to 2.5 ml an Amicon Ultra-15 concentrator unit (Millipore) with a molecular cut-off of 100 kDa and buffer exchanged to 150 mM NaCl, 20 mM Tris (pH 7.5), 0.5 mM EDTA, 10% glycerol and 0.01% $C_{12}E_8$ using PD-10 desalting column. The protein was further concentrated and loaded onto the Superdex 200 size exclusion chromatography (SEC) column in 150 mM NaCl, 20 mM Tris (pH 7.5), 0.5 mM EDTA, 10% glycerol and 0.01% $C_{12}E_8$.

BtuCD

The protein was purified following the same protocol as ModBC, except that protein was solubilized in 1% n-Dodecyl-N,N-Dimethylamine-N-Oxide (LDAO, Anatrace) and all the buffers were supplemented with 0.05% LDAO.

Native MS Experiments

Purified membrane proteins were buffer exchanged into MS Buffer (two times the CMC of detergent of interest and 200 mM ammonium acetate) using a centrifugal buffer exchange device (Micro Bio-Spin 6, Bio-Rad) as previously described (Laganowsky et al., 2013). The best quality mass spectra of all membrane proteins were obtained using 0.5% C_8E_4 (Anatrace) as detergent.

Soluble proteins were buffer exchanged into 200 mM ammonium acetate (Sigma Aldrich) using the same method. The freshly buffer-exchanged proteins were kept on ice, with protein concentration measured as before. The protein samples were diluted as desired in 200 mM ammonium acetate buffer with detergent as necessary and loaded into a gold-coated capillary Clark borosilicate capillary (Harvard Apparatus) prepared in the laboratory (Hernández and Robinson, 2007). The experiment was performed using a modified Q-Exactive hybrid quadrupole-Orbitrap mass spectrometer (Thermo Fisher Scientific, Bremen, Germany), optimised for analysing protein complexes of high mass and membrane proteins (Gault et al., 2016). The instrument was calibrated using caesium iodide solution. Typically, 2 μl of buffer exchanged protein solution was electrosprayed from gold-plated borosilicate capillaries

prepared in house. The instrument parameters for MS are: 1.2kV capillary voltage, S-lens RF 100%, quadrupole selection from 1,000 to 15,000 m/z range, collisional activation in the HCD cell 0-300 V, argon UHV pressure $1.2*10^{-9}$ mbar and 30 to 200°C capillary temperature, resolution of the instrument at 17,500 at m/z = 200 (a transient time of 64 ms) and ion transfer optics (injection flatapole, inter-flatapole lens, bent flatapole, transfer multipole: 8, 7, 6, 4 V respectively). The noise level was set at 3 rather than the default value of 4.64. Where required, baseline subtraction was performed to achieve a better-quality mass spectrum. Data were analysed using the Xcalibur 3.0 (Thermo Scientific) and UniDec (www.unidec.chem.ox.ac.uk) software packages. Theoretical and observed molecular masses of all species are described in Table 1.

Complex Formation Experiments

To allow the observation of BtuCD-F spectrum, it was necessary to incubate solutions of the two proteins in a 1:2 molar ratio (BtuF: BtuCD). In order to observe the effect of vitamin B_{12} on complex formation, 2 mM vitamin B_{12} (Sigma Aldrich) was added to a solution of BtuF before buffer exchange, then the solutions containing vitamin B_{12}-loaded BtuF and BtuCD were incubated and analysed as described above. Experiments with cyanocobinamide and vitamin B_9 (Sigma Aldrich) were performed in the same way as described above. To analyse the impact of ATP binding and hydrolysis, 5 mM ATP (Sigma Aldrich) and 5 mM $MgCl_2$ (Sigma Aldrich) were added to a solution of BtuCD before buffer exchange; then solutions containing BtuF and ADP-bound BtuCD were mixed and analysed as described above. The AMP-PNP experiments were performed by adding 5 mM AMP-PNP (Sigma Aldrich) to a solution of BtuCD before buffer exchange; then solutions containing BtuF and AMP-PNP-bound BtuCD were mixed and analysed as previously described. To assess the effect of both vitamin B_{12} binding and ATP binding and hydrolysis, 2 mM vitamin B_{12} was added to BtuF and 5 mM ATP was added to BtuCD before buffer exchange; then the solutions were mixed and analysed as mentioned above.

To detect the spectrum of ModBC-A in a 1:1 molar ratio was used (ModA:ModBC). Sodium molybdate (Na_2MoO_4, Sigma Aldrich) was added to a solution of ModA at a concentration of 5 mM, before buffer exchange. To analyse the influence of ATP-binding and hydrolysis, 5 mM ATP and 5mM $MgCl_2$ were added to a solution of ModBC before buffer exchange, then the solutions were incubated and analysed following the protocol described above. The spectrum obtained did not show ADP binding. The experiment was therefore repeated using the following method: to a solution of 7 μM ModBC in MS buffer, ATP and $MgCl_2$ were added to a final concentration of 10 μM. Then, MoO_4^{2-}-bound ModA and ADP-bound ModBC were incubated and analysed as described above.

Lipidomics Analysis

For LC-MS/MS analysis, the phospholipids were separated on a C18 column (Acclaim PepMap 100, C18, 75 μm × 15 cm; Thermo Scientific) by Dionex UltiMate 3000 RSLC nano System connected to a hybrid LTQ Orbitrap mass spectrometer (Thermo Scientific) via a dynamic nanospray source using PicoTip Emitter SilicaTip (New Objective). A binary buffer system was used with buffer A of ACN: H_2O (60:40), 10 mM ammonium formate, 0.1% formic acid and buffer B of IPA: ACN (90:10), 10 mM ammonium formate, 0,1% formic acid. The phospholipids were separated at 40°C with a gradient of 32% to 99% buffer B at a flow rate of 300 nl/min over 30 min. Typical MS conditions were: spray voltage of 1.8 kV and capillary temperature of 175°C. The LTQ-Orbitrap XL was operated in negative ion mode and in data-dependent acquisition with one MS scan followed by five MS/MS scans. Survey full-scan MS spectra were acquired in the Orbitrap (*m/z* 350−2,000) with a resolution of 60,000. Collision-induced dissociation (CID) fragmentation in the linear ion trap was performed for the five most intense ions at an automatic gain control target of 30,000 and a normalized collision energy of 38% at an activation of q = 0.25 and an activation time of 30 ms. Data were analysed by extracting ion chromatogram (XIC) and its area under the curve (AUC) of each cardiolipin was processed and integrated in Xcalibur3.0 (Thermo Scientific)) with 50 ppm 4 mass tolerance and 7 point Gaussian smoothing. The relative abundances of PEs and PGs in the samples were calculated based on the ratio of their AUCs to the sum of the AUCs of the phospholipids belonging to the same class.

QUANTIFICATION AND STATISTICAL ANALYSIS

All the experiments described in the paper were performed three times (*n*=3). Average values and standard deviations were calculated from at least 4 charge states in each of the three independent repeats and plotted using Microsoft Excel.

Structure, Volume *27*

Supplemental Information

The Different Effects of Substrates and Nucleotides on the Complex Formation of ABC Transporters

Francesco Fiorentino, Jani Reddy Bolla, Shahid Mehmood, and Carol V. Robinson

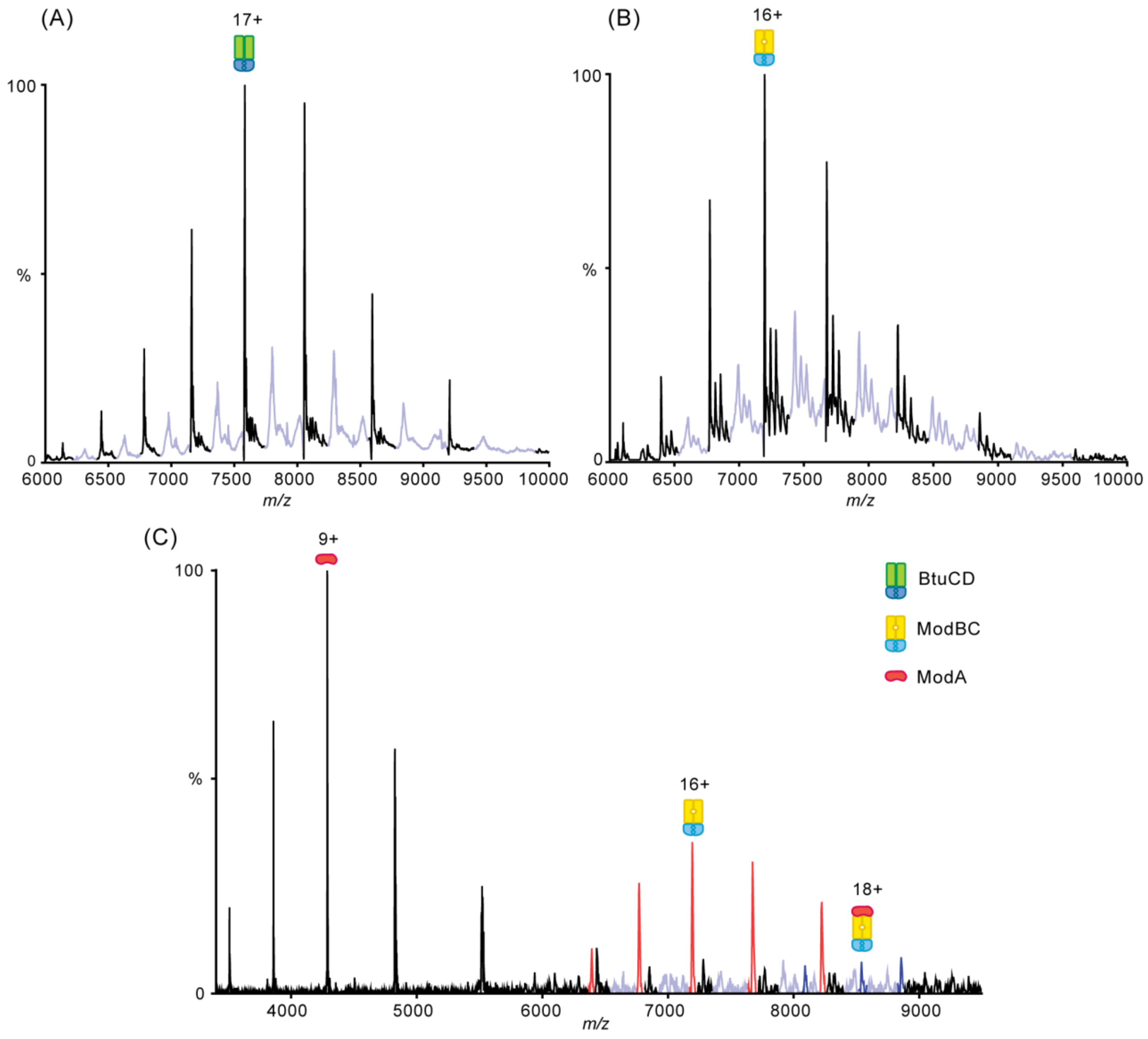

Figure S1. Mass spectra of uncomplexed BtuCD and ModBC, Related to Figure 1. (A) Mass spectrum of BtuCD in 0.50% (w/v) C_8E_4 reveals a charge state series consistent with the tetrameric transporter with different lipid-bound species, mostly LPS (light blue peaks). (B) Mass spectrum of ModBC in 0.50% (w/v) C_8E_4 reveals a charge state series consistent with the tetrameric transporter bound to different lipid species, mainly LPS (light blue peaks). (C) Mass spectrum of ModBC and ModA acquired in the absence of molybdate shows only a low population of the full ModBC-A complex (blue peaks).

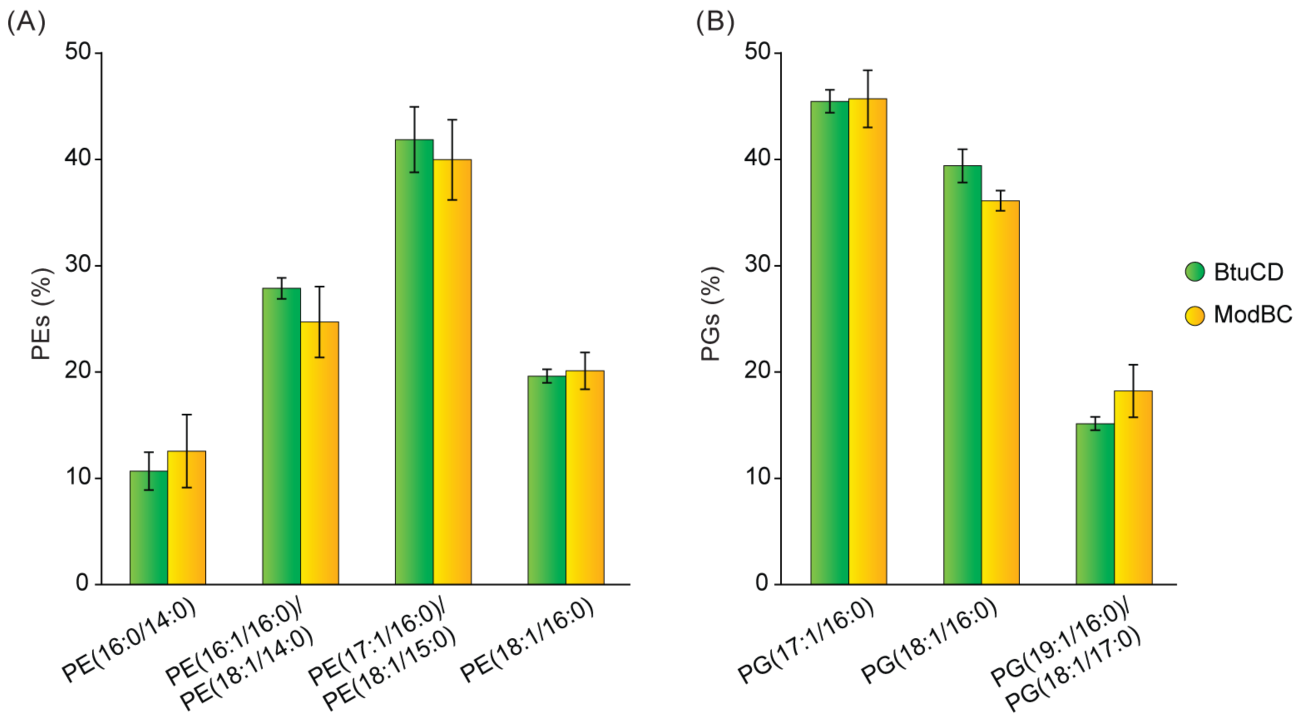

Figure S2. Lipidomics analysis for BtuCD (green) and ModBC (yellow), Related to Figure 1. (A) Relative abundance of phosphatidylethanolamines (PEs). (B) Relative abundance of phosphatidylglycerols (PGs). Error bars represent standard deviations (*n*=3).

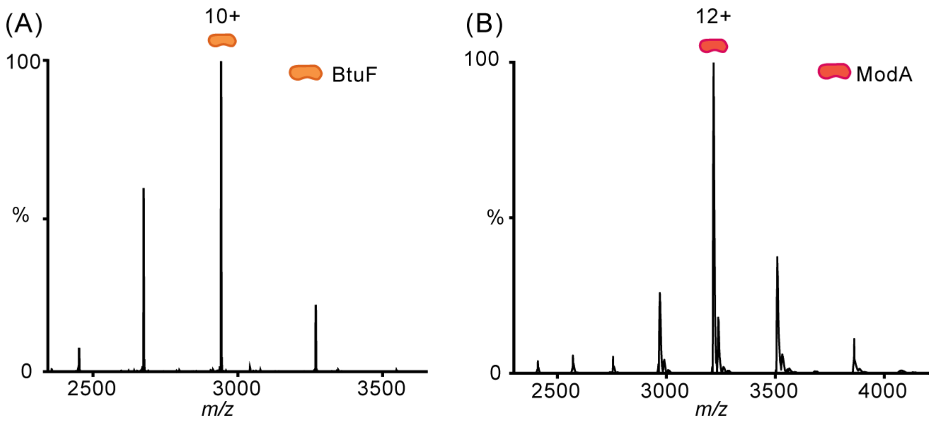

Figure S3. Mass spectra of BtuF (A) and ModA (B), Related to Figure 1. Both proteins were separately purified and analysed in the absence of any detergent. Theoretical and observed masses and recorded in Table 1.

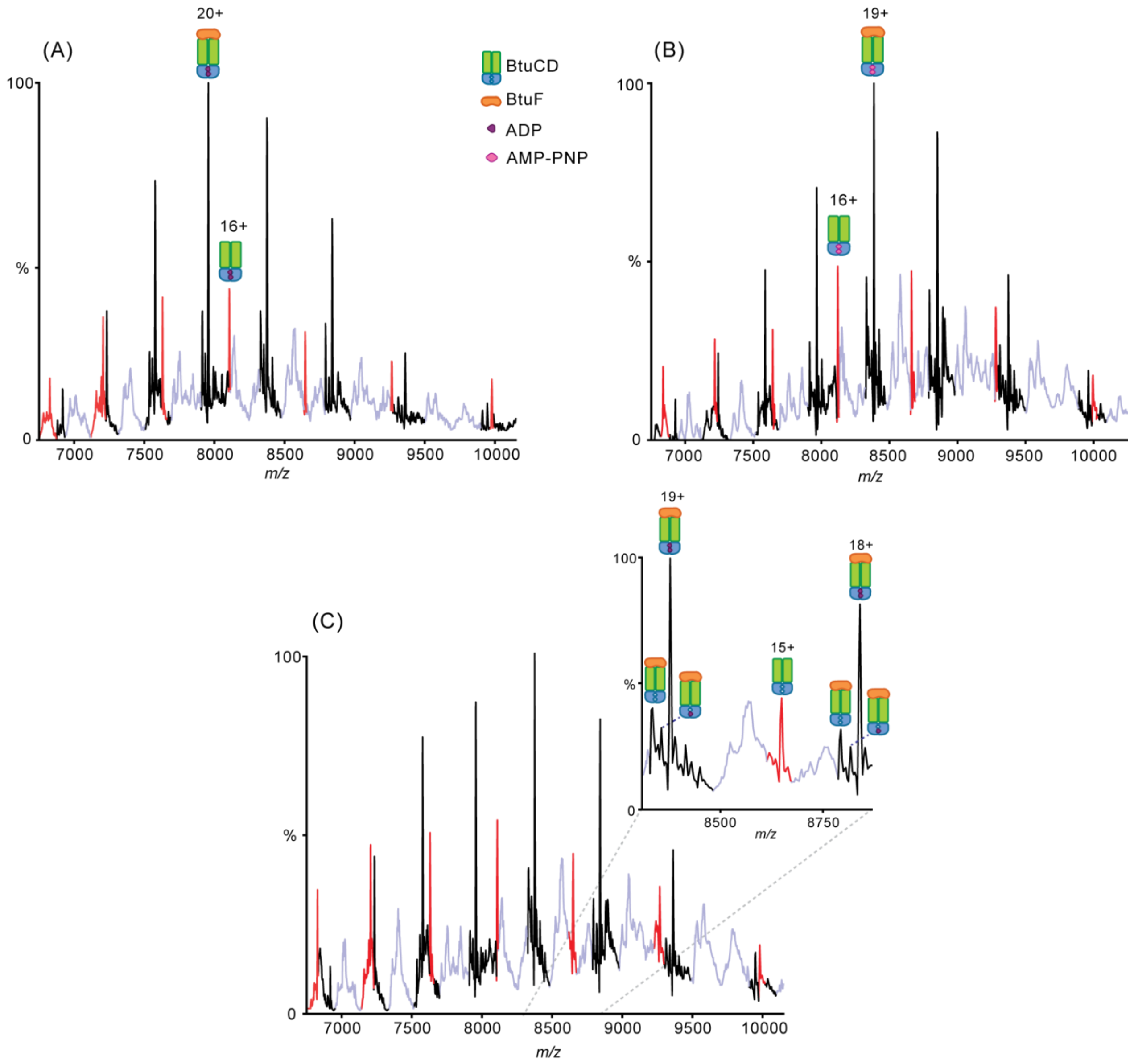

Figure S4. Nucleotides destabilise BtuCD-BtuF interaction, Related to Figure 3. (A) Mass spectrum of BtuCD-F recorded after addition of 5 mM ATP to BtuCD before buffer exchange. ADP-bound species are detected with binding indicative of cooperativity along with an increase in the intensity of the BtuCD charge states (red peaks). (B) Mass spectrum of BtuCD-F recorded after addition of 5 mM AMP-PNP to BtuCD before buffer exchange. Also in this case, AMP-PNP-bound species are detected with binding indicative of cooperativity along with an increase in the intensity of the BtuCD charge states (red peaks). (C) Mass spectrum of BtuCD-F recorded after addition of 5 mM ATP to BtuCD and 2mM vitamin B_{12} to BtuF before buffer exchange. The spectrum shows a further increase in the BtuCD charge states evident of a synergistic effect upon the transport of B_{12} and the binding of nucleotides (red peaks).

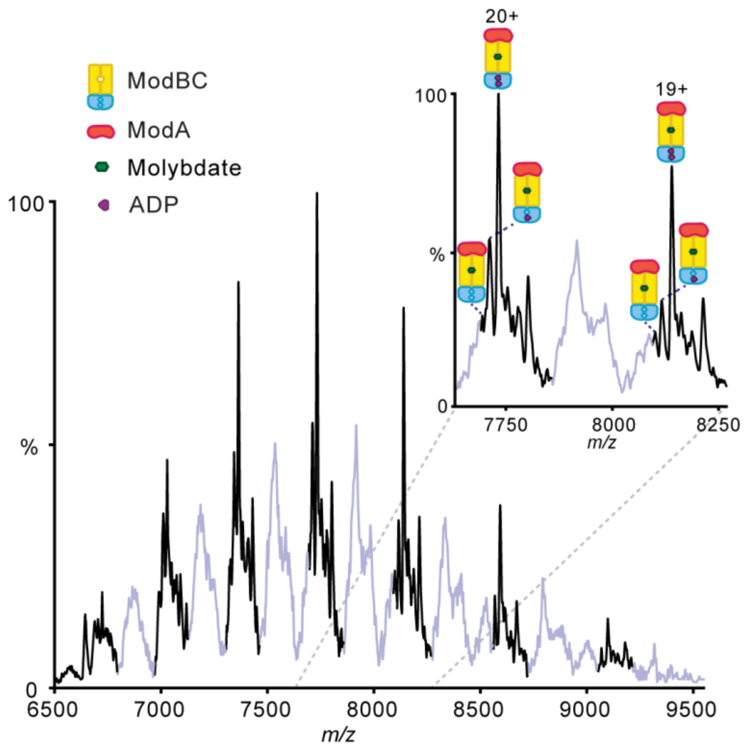

Figure S5. Mass spectrum of ADP-bound form of ModBC-A, Related to Figure 3. Mass spectrum of ModBC-A following the addition of 10 µM ATP to ModBC after buffer exchange.
